# Measuring safety climate in acute hospitals: Rasch analysis of the safety attitudes questionnaire

**DOI:** 10.1186/s12913-016-1744-4

**Published:** 2016-09-20

**Authors:** Sze-Ee Soh, Anna Barker, Renata Morello, Megan Dalton, Caroline Brand

**Affiliations:** 1 Department of Epidemiology and Preventative Medicine, Monash University, Melbourne, VIC Australia; 2 Department of Physiotherapy, Alfred Health, Melbourne, VIC Australia; 3 Department of Physiotherapy, Monash University, Melbourne, VIC Australia; 4 Faculty of Medicine, Nursing and Health Sciences, Monash University, Melbourne, VIC Australia; 5 School of Human, Health and Social Science, Central Queensland University, Rockhampton, QLD Australia; 6 Melbourne EpiCentre, University of Melbourne and Melbourne Health, Melbourne, VIC Australia

**Keywords:** Safety culture, Patient safety, Rasch analysis

## Abstract

**Background:**

The Safety Attitudes Questionnaire (SAQ) is commonly used to assess staff perception of safety climate within their clinical environment. The psychometric properties of the SAQ have previously been explored with confirmatory factor analysis and found to have some issues with construct validity. This study aimed to extend the psychometric evaluations of the SAQ by using Rasch analysis.

**Methods:**

Assessment of internal construct validity included overall fit to the Rasch model (unidimensionality), response formats, targeting, differential item functioning (DIF) and person-separation index (PSI).

**Results:**

A total of 420 nurses completed the SAQ (response rate 60 %). Data showed overall fit to a Rasch model of expected item functioning for interval scale measurement. The questionnaire demonstrated unidimensionality confirming the appropriateness of summing the items in each domain. Score reliabilities were appropriate (internal consistency PSI 0.6–0.8). However, participants were not using the response options on the SAQ in a consistent manner. All domains demonstrated suboptimal targeting and showed compromised score precision towards higher levels of safety climate (substantial ceiling effects).

**Conclusion:**

There was general support for the reliability of the SAQ as a measure of safety climate although it may not be able to detect small but clinically important changes in safety climate within an organisation. Further refinement of the SAQ is warranted. This may involve changing the response options and including new items to improve the overall targeting of the scale.

**Trial registration:**

This study was registered with the Australian New Zealand Clinical Trials Registry, number ACTRN12611000332921 (21 March 2011).

**Electronic supplementary material:**

The online version of this article (doi:10.1186/s12913-016-1744-4) contains supplementary material, which is available to authorized users.

## Background

The Institute of Medicine has identified that gaps in organisational culture may contribute to suboptimal patient safety [1]. Consequently, there has been a growing trend for healthcare organisations to measure patient safety culture. Safety culture refers to *“the product of individual and group values, norms, attitudes, beliefs, perceptions, competencies and the patterns of behaviour that determine the commitment to…an organisation’s health and safety management”* (pg ii18) [2]. There are numerous challenges associated with defining the measurable components of safety culture [3]. Safety climate, which has been described as the shared perceptions, attitudes and beliefs of employees about the way in which a hospital manages and achieves patient safety [3, 4], has been used to provide a snapshot of the safety culture of an organisation.

Numerous questionnaires have been developed to quantify safety climate [4–6]. One of the most frequently evaluated and widely used is the Safety Attitudes Questionnaire (SAQ) that assesses six safety-related climate domains including teamwork climate; job satisfaction; perceptions of management; safety climate; working conditions; and stress recognition [7]. It has been translated into a variety of languages and used in different countries including Taiwan, Norway, Brazil, Germany and Sweden [8–13]. To date no study has validated the SAQ for use in Australian hospitals.

The SAQ has previously been shown to have good internal consistency, test re-test reliability and predictive validity [7–9, 11, 12, 14]. The factor structure of the SAQ has also been tested with confirmatory factor analysis (CFA) [9, 11, 13]. Whilst the construct validity of the questionnaire can be considered to be acceptable, some studies showed a degree of misfit with the CFA model [9, 12]. This suggests that some items in the SAQ may not be measuring the same underlying safety climate construct. For instance, the stress recognition domain does not correlate strongly with other SAQ domains [15, 16]. This may have implications on the overall validity of the SAQ as it is unclear what construct the stress recognition scores are measuring [15–17]. Further psychometric evaluation is therefore required to provide greater detail on the measurement properties of the SAQ. This will inform its use in quantifying the perceived climate of patient safety in a specific clinical environment.

Rasch analysis is a modern psychometric approach based on latent-trait modelling that allows examination of key measurement and scaling properties of an outcome tool [18]. Rasch modelling enables the conversion of equal units of measurement from raw (ordinal data) scores on items of a questionnaire to interval-level scores [19]. It also provides an opportunity to examine the unimensionality of domains (i.e. measurement of one underlying construct), ceiling and floor effects and whether or not items are ‘biased’ for specific groups for example based on clinical speciality (differential item functioning [DIF]) [18]. As such it is argued that the Rasch measurement model is the standard for evaluating the psychometric properties of scales. This is despite the limitation of Rasch analysis which requires a large number of observations to estimate the parameters of the model [20].

This study aimed to extend the psychometric evaluations of the SAQ Short Form [7] by examining the internal construct validity of the questionnaire using Rasch analysis in the Australian context. This would allow us to: (1) examine the unidimensionality of the six SAQ domains; (2) investigate the response formats of the questionnaire; (3) assess whether the six SAQ domains are appropriately targeted for the clinical population (floor and ceiling effects); (4) examine the extent to which items distinguish between different levels of safety climate; and (5) assess whether different groups within the sample (e.g. medical versus surgical wards), despite equal levels of the underlying characteristics being measured, respond in a different manner to an individual item.

## Methods

### Participants and setting

The sample for this analysis was derived from nurses that completed the SAQ as part of the 6-PACK cluster randomised clinical trial (RCT). The sample and sampling procedures for the 6-PACK project, including hospital and ward selection, have been described in detail elsewhere [21]. In brief, six public hospitals in metropolitan and regional Victoria, and metropolitan New South Wales, Australia agreed to participate in the RCT. Hospitals ranged in size from moderate (200–500 bed) to large (>500 beds). Each hospital identified acute surgical and medical wards where the average length of stay was less than 10 days, where falls commonly occurred, and had low levels of use of the falls prevention strategies being tested in the 6-PACK project. Sixteen medical and eight surgical wards were included in the RCT.

Nurses were invited to complete the SAQ if they had worked on the participating wards for more than 7.5 h per week in the two months prior to the survey being administered. Staff that did not meet the above criteria were excluded from this study because they might have limited knowledge of, or exposure to the ward (and hospital) culture. If nurses completed and returned the survey, it was assumed that they agreed and consented to participate in this study. The SAQ was administered to 702 nurses from the 24 acute wards.

### The SAQ

The SAQ is a refinement of the Intensive Care Unit Management Attitudes Questionnaire, which in turn was derived from the Flight Management Attitudes Questionnaire [7]. The original version of the SAQ consists of 60 items, with 30 core items that are identical in all clinical settings [12]. A short form version of the SAQ that included the 30 core items and six additional items of interest to senior hospital leaders was used in this study to measure safety climate [7, 12]. The SAQ Short Form assesses six-safety related climate domains including teamwork climate (6 items); job satisfaction (5 items), perceptions of management (6 items); safety climate (7 items); working conditions (4 items); and stress recognition (3 items) [7]. Five of the items in the questionnaire are responded to separately for the hospital and ward unit, yielding a total of 41 items [7, 12]. The SAQ Short Form has been used to compare safety climate within and between facilities, and benchmarking data is available to allow organisations to evaluate their own climate data [7].

As per Gallego et al. [15], the SAQ Short Form was modified slightly in this study to reflect the Australian hospital workplace, e.g. substituting ‘clinical area’ for ‘ward’. The SAQ items were also combined with questions relating to staff knowledge and perceptions of falls prevention strategies (see Additional file 1). Whilst there may be a possibility that these additional questions may influence responses to the SAQ items, the participant and logistical burden of administering two different questionnaires outweighed this risk. Responses for the SAQ items were recorded using a 5-point numeric scale to reflect the level of agreement with each individual item. Scores within each domain were calculated and converted to a 100-point numeric scale [7]. Higher scores indicate greater agreement that more positive attitudes exist towards the particular safety domain assessed.

### Statistical analysis

All data were analysed using SPSS v22.0 (SPSS Inc., Chicago, Illinois) with the Rasch analysis, being completed using the RUMM2030 package using a partial credit model (RUMM Laboratory Pty Ltd, Perth, Australia). The methods and criteria for assessing the measurement properties of the SAQ using Rasch analysis are outlined in Table 1.

**Table 1:** Statistical tests and criteria for assessment to examine specific measurement properties of the SAQ

Measurement property	Purpose	Statistical test	Criteria for assessment
Unidimensionality	To assess whether items in each SAQ domain measure a single construct (or concept)	• Residual fit statistics • Item-fit residuals • Person-fit residuals	• A fit residual SD value >1.5 would suggest a problem [18]. • Item residuals that range between −2.5 and 2.5 indicate adequate fit to the model [18].^a^
Response formats (thresholds)	To assess whether participants had difficulty discriminating between the response options on the SAQ	• Threshold map • Category probability curves	• Pattern of thresholds examined. • Ordering of thresholds where each response category systematically take turns to be the most likely response [18].
Targeting	To assess whether the 6 SAQ domains are appropriately targeted for the clinical population (floor and ceiling effects)	• Mean location score • Person-item threshold distribution map	• The mean sample location should approximate the mean item location (i.e. zero) for a well-targeted measure [18].
Internal consistency reliability	To assess the extent to which items distinguish between levels of safety climate	• Person separation index (PSI)	• A PSI of a > 0.7 indicates the items of the scale is able to separate the participants in the sample [24].^b^
Item bias	To assess whether different groups within the sample (e.g. medical or surgical ward and nursing qualification), despite equal levels of the underlying characteristic being measured, responds in a different manner to an individual item	• Differential item functioning (DIF)	• Uniform DIF is indicated by a significant main effect for the person factor (e.g. ward type) [18]. • Non-uniform DIF is indicated by a significant interaction effect [18].

^a^Items with large negative residual values indicate a high level of predictability in responses and signal possible item redundancy. Items with large positive residual values suggest an item does not contribute to the measurement of a unidimensional construct

^b^A PSI is the same as Cronbach’s alpha with the logit value replacing the raw score in the same formulae

### Sample size

In order to obtain an accurate estimation from the Rasch analysis, it is generally recommended that a minimum of ten categories per response option is available to ensure that responses are appropriately distributed across the response categories [22]. The sample size required for analysis also depends on whether items in the scale are targeted properly to participants in the sample [18]. If the scale is well-targeted, a sample size of 108 will be required for accurate estimation but if it is not, a sample of 243 will be needed [23]. Given that a sample of 420 participants was available in this study, an appropriate degree of precision can be expected from the Rasch analysis of the SAQ [18].

## Results

### Participant characteristics

The final sample for this analysis included 420 nurses from the 24 acute wards (response rate 60 %). Additional file 2 illustrates the characteristics of nurses that completed the SAQ. The majority of respondents were registered nurses (74 %) working on a medical ward (75 %) with at least one or more years of experience (74 %). Most of the nurses also worked more than two shifts per week (93 %). On average, 53 % of nurses held positive attitudes towards job satisfaction, followed by teamwork climate (51 %) and safety climate (41 %). In contrast, only 9 % of nurses across all six hospitals responded favourably towards perceptions of hospital management. A degree of variability was observed in the percentage of staff with a positive safety climate across the six hospitals. Hospital Two and Five appeared to report lower levels of teamwork climate, safety climate, perceptions of ward management and working conditions.

### Overall model fit, unidimensionality and reliability

Results of the Rasch analyses are summarised in Table 2. The *χ*2 Item-Trait Interaction statistic indicates good overall fit between the data and the Rasch measurement model for the domains of safety climate, stress recognition and working conditions. The remaining SAQ domains of teamwork climate, job satisfaction and perceptions of ward and hospital management were found to have some degree of misfit between the data and the model (*χ*^2^ 59.2–164.6; *p* < 0.01). This is likely due to a deviation between an individual item and the rest of the items in the domain as the fit residual standard deviation (SD) values were above 1.5 (SD 1.65–4.02), much higher than the expected value of 1 [18].

**Table 2:** Overall Rasch model fit statistics and reliability of SAQ domains^a^

Overall fit statistics	SAQ domains						
	Teamwork climate	Safety climate	Job satisfaction	Stress recognition	Perceptions of ward management	Perceptions of hospital management	Working conditions
Items							
Fit residual (mean)^b^	0.46	−0.03	−0.44	0.32	0.31	0.15	−0.14
Fit residual (SD)^c^	1.65	0.86	2.10	1.49	3.92	4.02	1.17
Persons							
Fit residual (mean)^b^	−0.58	−0.61	−0.52	−0.50	−0.64	−0.98	−0.79
Fit residual (SD)^c^	1.37	1.40	1.11	1.13	1.39	1.99	1.37
Total item-trait interaction							
Total item *χ* ^2^	70.88	40.35	59.16	41.37	164.61	153.20	14.17
d*f*	36	42	30	24	36	36	15
*p*-value	0.00	0.54	0.00	0.02	0.00	0.00	0.51
Person separation index^d^	0.71	0.74	0.80	0.73	0.66	0.67	0.61

*SD* standard deviation, d*f* degrees of freedom

^a^As analysed using RUMM2030 (Rumm Laboratory Pty Ltd., Perth) for Windows

^b^Should be close to 0 [18]

^c^Should be close to 1 [18]

^d^Rasch based reliability statistic (analogous to Cronbach’s alpha)

Analysis of individual item fit statistics revealed that there were items in the teamwork climate, job satisfaction and perceptions of ward and hospital management domains that deviated significantly from the Rasch model (Additional file 3). Misfitting items or persons were indicated by two statistics: a fit residual value beyond ±2.5 or a significant chi-square probability value [18, 24]. Items with large negative residual values (items 18, 24, 26) indicate a high level of predictability in responses and signal possible item redundancy. Items with large positive residual values (items 2, 25, 29) indicate an item does not contribute to the measurement of the same underlying construct [25]. This suggests that some items in the teamwork climate and perceptions of management domains may not be measuring the same construct as other items within the domain [18].

Despite this, all six domains of the SAQ demonstrated unidimensionality. Responses to items in each domain were not dependent on the response to another item. Principal Components Analysis (PCA) on the residuals further supported unidimensionality because items in each SAQ domain did not measure different aspects of the underlying construct such as teamwork climate, safety climate and job satisfaction (paired *t* test <0.05). This provides some support to the appropriateness of adding up items in each domain [26].

The person separation index (PSI) statistic for the six SAQ domains ranged from 0.66 to 0.80 (Table 2), indicating that most domains had good internal consistency reliability [18]. The exception was the working conditions domain which had a PSI value of 0.61. This result may have been influenced by the small number of items (*n* = 3) in this domain [27].

### Response formats

**Fig. 1:**
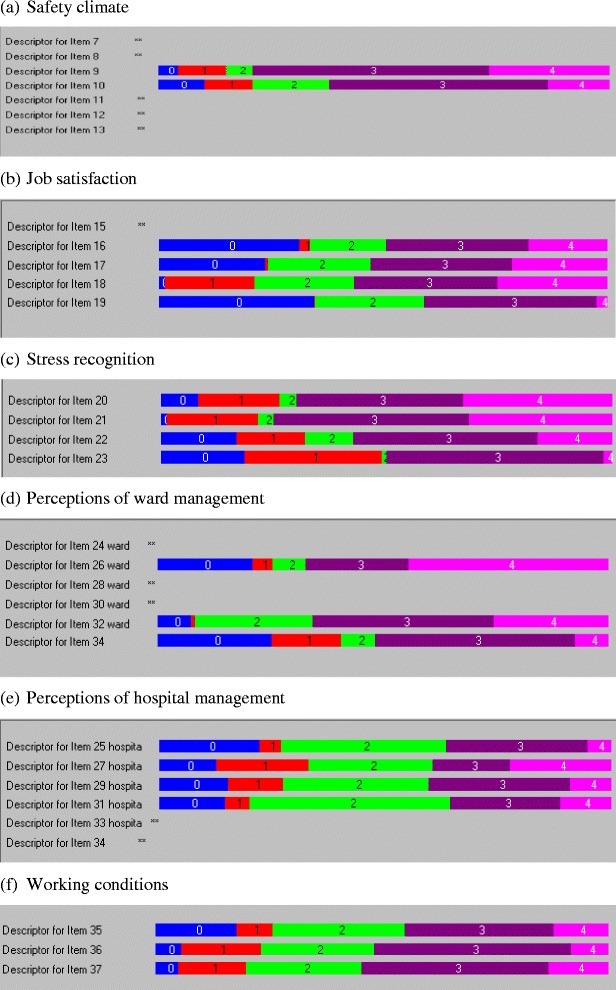
Threshold maps for SAQ domains. Note that as disordered thresholds were observed for all six items in the Teamwork Climate domain, a threshold map was not generated

As shown in Fig. 1, disordered thresholds were observed for all SAQ domains except working conditions. This indicates that participants were not using the response options on the SAQ (strongly disagree to strongly agree) in a consistent manner. In particular, participants appeared to have difficulty distinguishing between the strongly disagree and disagree response options for the majority of items.

### Targeting

**Fig. 2:**
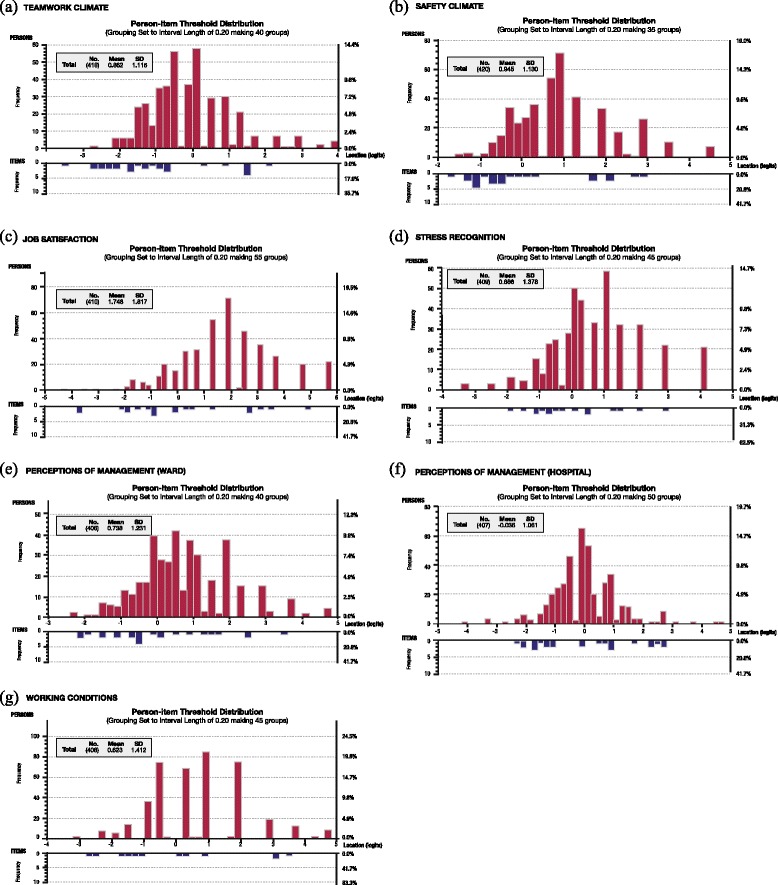
Person-item threshold distribution depicting targeting for the six domains of the SAQ. Distributions of the locations of people and items on the common logit metric (negative values = poor safety climate; positive values = good safety climate) are depicted on the upper and lower panels respectively

Inspection of the relationship between the distributions of persons relative to items indicated that all six SAQ domains were poorly targeted. As shown in Fig. 2, there were insufficient items to assess the full range of safety climate in this sample of nurses across all six domains [18]. Floor and ceiling effects were observed for the job satisfaction, perceptions of hospital management and stress recognition domains. The domains perceptions of ward management, safety climate, teamwork climate and working conditions displayed ceiling effects (Fig. 2). This sample of nurses also had higher levels of safety climate on average for all domains (mean location scores range 0.63 to 1.76) except the perception of hospital management domain (mean location score −0.04).

### Item bias

The possibility of differences in responses to the SAQ items based on whether nurses were working on a medical or surgical ward was explored by analysis of DIF. Items 7 (safety climate domain) and 28 (perceptions of ward management domain) showed some degree of uniform DIF (*p* = 0.000). In contrast, the only item that displayed significant non-uniform DIF by ward type was item 28 in the perceptions of hospital management domain (*p* = 0.000). This suggests that the differences in the responses between nurses working on medical and surgical wards were not consistent.

## Discussion

Given the emerging evidence linking positive safety climate with improvements in patient safety outcomes [28, 29], there has been a reliance on safety climate questionnaires to identify specific wards or units with low levels of safety climate to guide the implementation of strategies to improve safety culture. However, little is known about whether a safety climate questionnaire such as the SAQ adequately measures the safety culture of an organisation or clinical unit [30]. This study provides new information about the internal construct validity of the SAQ using Rasch analysis in the Australian context. It is one step towards increasing the understanding of safety climate in the measurement of patient safety culture. The Rasch measurement model is recognised as the gold standard for psychometric evaluations of outcome scales [20]. It has been recommended that Rasch analysis is used during the development phase or when reviewing the psychometric properties of existing questionnaires [20, 31]. Findings from this study can be used to inform the refinement of the SAQ to improve its psychometric properties in order to accurately measure safety climate in clinical environments.

All six SAQ domains demonstrated unidimensionality and the responses to items in each domain were not dependent on another item. This provides support for summing the items in each domain [20]. Nevertheless, there were some items in the teamwork climate and perceptions of management domains that may not measure the same underlying construct, as indicated by positive item fit residual values greater than 2.5. In addition, there were potentially some redundant items in the job satisfaction and perceptions of management domains. The presence of disordered thresholds may have affected the fit of these individual items because participants had difficulty distinguishing between the strongly disagree and disagree response options [18]. The option of a midpoint ‘neutral’ category in the SAQ may have also contributed to the disordered thresholds. It may be worthwhile for future studies to use Rasch analysis to examine whether changing the response options (for example, removing the neutral option) or removing the redundant items (for example, items 18, 24 and 26) may improve the overall model fit of the SAQ. This may improve the ability of the SAQ to distinguish between different levels of safety climate in a clinical setting.

All six domains of the SAQ appeared to have suboptimal targeting. This is particularly evident in the Rasch analyses of the person and item distributions, where all domains demonstrated substantial ceiling effects (Fig. 2). The lack of measurement precision observed may be due to sampling effects because targeting relates to the characteristics of the investigated sample [18]. The inclusion of only nurses in this study may have also contributed to the floor and ceiling effects observed. Further investigations using other health professionals including hospital executives would be beneficial in order to determine whether the level of safety climate assessed by the SAQ is consistent with staff working in the clinical environment.

Floor effects or low levels of safety climate were also not represented in the job satisfaction, perceptions of management and stress recognition domains. This finding may have implications on how the SAQ can be used as a tool to quantify the levels of safety climate in an organisation as it may not be able to detect small but clinically important changes in safety climate. Given the need for accurate measurement tools to drive improvement in patient safety and optimise resource allocation [32], further refinement of the SAQ is warranted. This may involve rewording existing items in order to improve the measurement of safety climate at either ends of the scale. It may also be beneficial to include items from other safety climate questionnaires such as the Hospital Survey on Patient Safety Culture (HSOPS) [33]. This may improve the overall targeting of the SAQ as the item pool is expanded through the use of an item bank [32, 34], which allows a set of items that measures a single construct to be selected without substantial loss of measurement precision [34].

### Strengths and limitations

One of the strengths of this study was that it was a multi-centre design and included 420 nurses working across 24 acute hospital wards. Additionally, the sample for analysis had worked on the participating wards for a substantial period of time. Most nurses had worked within the organisation for at least one year and had more than two shifts a week. This means that they were aware and conscious of the level of safety climate on the ward. However, the limitations of this study must also be considered. Firstly, participants were nurses working in acute hospitals. We did not include other health professionals such as doctors and allied health staff, which markedly limits the generalisability of the safety climate findings. Secondly, questions regarding falls prevention strategies in the acute setting were combined with the SAQ items. These additional questions were closely related to patient safety and may have affected how nurses responded to the SAQ questions. They may have been more aware of how the ward may or may not be managing patient safety, which may explain the lower levels of safety climate observed in this sample. There is also a potential the sample may be biased towards wards with lower levels of safety climate as it included wards where ‘falls commonly occurred’ and had ‘low levels of use of falls prevention strategies’ [21]. There may be less nurses in this sample with a positive safety climate attitude compared to the general population. This may have implications on the precision of the estimates from the Rasch model, particularly with respect to targeting and item difficulty. Finally, caution is required when interpreting the results for item bias because differences in responses to the SAQ were not examined based on the age or gender of nurses working in the acute wards.

### Recommendations

The SAQ has demonstrated adequate internal consistency reliability as a measure of safety climate in acute Australian hospital. It is also appropriate to sum items in each domain without weighting or standardisation. The results of the Rasch analysis, however, suggest that further refinement of some items and response options may be warranted in order to minimise the floor and ceiling effects and improve overall model fit. This may involve rewording existing items and including new items to accurately measure small but clinical meaningful changes in safety climate. We also recommend that further validation work of the SAQ be undertaken in different settings and amongst different health professionals in order to improve our understanding of safety climate in the measurement of safety culture in Australian hospitals. This includes examining the variability in safety climate across hospitals and whether these differences may be associated with the incidence of patient safety outcomes such as falls, pressure injuries and medication errors.

## Conclusion

This is the first validation study of the SAQ using the Rasch measurement model and has provided important insights into the internal construct validity of the SAQ. We found some limitations associated with some items not measuring the same underlying construct as well as substantial floor and ceiling effects. This may limit the ability of the questionnaire to precisely measure the underlying levels of safety climate in a clinical environment. Additional research is needed to refine the SAQ. Further studies linking levels of safety climate with patient safety outcomes including falls and fall-related injuries also warrants further investigation.

## Additional files

Additional file 1: Safety climate survey administered to nursing staff (DOCX 41 kb) Additional file 2: Characteristics of nurses that completed the Safety Attitudes Questionnaire (SAQ) by hospital (DOCX 17 kb) Additional file 3: Rasch item and fit statistics for the Safety Attitudes Questionnaire (SAQ) (DOCX 18 kb)

## Abbreviations

CFA: Confirmatory actor analysis
DIF: Differential item functioning
PCA: Principal components analysis
PSI: Person separation index
RCT: Randomised controlled trial
SAQ: Safety attitudes questionnaire
SD: Standard deviation
SE: Standard error
