# Supplementary material for: Measuring safety climate in acute hospitals: Rasch analysis of the safety attitudes questionnaire
Source: BMC Health Serv Res. 2016 Sep 20;16:497. doi: 10.1186/s12913-016-1744-4 (PMC5029072; doi:10.1186/s12913-016-1744-4)
Supplement: Additional file 1: — Safety climate survey administered to nursing staff (DOCX 41 kb) [file 12913_2016_1744_MOESM1_ESM.docx]

**NURSE SURVEY**

**Thank you for participating in this survey**

# We recognise the importance of your privacy so please note:

- *All information collected in this survey will be anonymous.*
- *Your employer will not know if you have participated in the survey.*
- *No personal details are required so we have no way of linking your survey response to you.*
- *All results will be grouped for example “80% of nurses strongly agreed that falls risk assessment tools are a useful way of identifying patients at risk of falling”.*

## Please answer the following five questions by ticking the appropriate box:

1. How long have you worked at this hospital?

- <4 months □ 4-12 months □ 1-5 years □ > 5 years

1. How long have you worked on this ward?

- <4 months □ 4-12 months □ 1-5 years □ > 5 years

1. What is your qualification?

- Registered nurse □ AIN □ Other (specify)

1. On what ward do you most frequently work?

□ 9E □ 6E □ 11W □ 7E1

1. How many shifts do you usually work on the above ward?

- <1 shift per week □ 1 shift per week □ 2-4 shifts per week □ 5 shifts per week

Please circle the response that best matches your perceptions/experiences of the falls prevention and safety climate **on the ward where you most frequently work**.

| A | B | C | D | E |
| --- | --- | --- | --- | --- |
| Strongly disagree | Disagree | Neutral | Agree | Strongly agree |

| **Please answer the following items with respect to your ward using the scale below.** | | | | | Strongly agree▼ | | | | |
| --- | --- | --- | --- | --- | --- | --- | --- | --- | --- |
|  | | | | Agree▼ | | | | |  |
|  | | | Neutral▼ | | | | |  |  |
|  | | Disagree▼ | | | | |  |  |  |
|  | Strongly disagree▼ | | | | |  |  |  |  |
| Nurse input is well received on this ward. | | | | | A | B | C | D | E |
| The current falls prevention program is effective at reducing falls on my ward. | | | | | A | B | C | D | E |
| In this ward, it is difficult to speak up if I perceive a problem with patient care. | | | | | A | B | C | D | E |
| Falls risk assessment tools are a useful way of identifying patients at risk of falling. | | | | | A | B | C | D | E |
| Disagreements in this ward are resolved appropriately (i.e. not who is right, but what is best for the patient). | | | | | A | B | C | D | E |
| Falls risk assessment tools are better than my own judgment for identifying patients most at risk of falling. | | | | | A | B | C | D | E |
| I have the support I need from other staff to care for patients. | | | | | A | B | C | D | E |
| Low-low beds are an effective way to prevent injuries in patients at risk of falling out of bed. | | | | | A | B | C | D | E |
| It is easy for personnel here to ask questions when there is something that they do not understand | | | | | A | B | C | D | E |
| Keeping the bed rails up is an effective way to prevent injuries in patients at risk of falling out of bed. | | | | | A | B | C | D | E |
| The physicians and nurses in this ward work together as a well-coordinated team. | | | | | A | B | C | D | E |
| It is not my responsibility to stop patients from falling. | | | | | A | B | C | D | E |
| I would feel safe being treated here as a patient. | | | | | A | B | C | D | E |
| Falls risk assessment is a waste of time. | | | | | A | B | C | D | E |
| Medical errors are handled appropriately in this ward. | | | | | A | B | C | D | E |
| The falls risk assessment tool used on this ward is a useful way of identifying patients at risk of falling. | | | | | A | B | C | D | E |
| I know the proper channels to direct questions regarding patient safety in this ward. | | | | | A | B | C | D | E |
| I don’t have time to complete a falls risk assessment on all of my patients. | | | | | A | B | C | D | E |
| I receive appropriate feedback about my performance. | | | | | A | B | C | D | E |
| A “Falls risk” sign above the bed is a useful way to communicate to staff what patients are at risk of falling. | | | | | A | B | C | D | E |

| A | B | C | D | E |
| --- | --- | --- | --- | --- |
| Strongly disagree | Disagree | Neutral | Agree | Strongly agree |

| **Please answer the following items with respect to your ward using the scale below.** | | | | | | | | | | | Strongly agree▼ | | | | | |
| --- | --- | --- | --- | --- | --- | --- | --- | --- | --- | --- | --- | --- | --- | --- | --- | --- |
|  | | | | | | | | | | Agree▼ | | | | | |  |
|  | | | | | | | | | Neutral▼ | | | | | |  |  |
|  | | | | | | Disagree▼ | | | | | | | |  |  |  |
|  | | | | Strongly disagree▼ | | | | | | | | |  |  |  |  |
| My suggestions about safety would be acted upon if I expressed them to management on this ward. | | | | | | | | | | | | A | B | C | D | E |
| It is my responsibility, as a patient’s treating nurse, to assess their falls risk each shift. | | | | | | | | | | | | A | B | C | D | E |
| I like my job. | | | | | | | | | | | | A | B | C | D | E |
| It is my responsibility to implement prevention strategies for patients I identify as high falls risk. | | | | | | | | | | | | A | B | C | D | E |
| Working on this ward is like being part of a large family. | | | | | | | | | | | | A | B | C | D | E |
| Falls are not a problem on my ward so falls prevention programs are not required. | | | | | | | | | | | | A | B | C | D | E |
| This ward is a good place to work. | | | | | | | | | | | | A | B | C | D | E |
| Falls prevention is not a priority on this ward. | | | | | | | | | | | | A | B | C | D | E |
| I am proud to work on this ward. | | | | | | | | | | | | A | B | C | D | E |
| Incident reporting provides us with a way of measuring how we are going with patient falls. | | | | | | | | | | | | A | B | C | D | E |
| Morale amongst staff on this ward is high. | | | | | | | | | | | | A | B | C | D | E |
| I never know what to write on a falls incident report. | | | | | | | | | | | | A | B | C | D | E |
| When my workload becomes excessive, my performance is impaired. | | | | | | | | | | | | A | B | C | D | E |
| I know incident reporting is important but I just don’t have time to do it. | | | | | | | | | | | | A | B | C | D | E |
| I am less effective at work when fatigued. | | | | | | | | | | | | A | B | C | D | E |
| I only report falls in which the patient suffers an injury. | | | | | | | | | | | | A | B | C | D | E |
| I am more likely to make errors in tense or hostile situations. | | | | | | | | | | | | A | B | C | D | E |
| Falls prevention is primarily the responsibility of the physiotherapist. | | | | | | | | | | | | A | B | C | D | E |
| Fatigue impairs my performance during emergency situations (e.g. emergency resuscitation, seizure). | | | | | | | | | | | | A | B | C | D | E |
| Management supports my efforts: | Unit Mgt | A | B | C | D | | E | Hosp Mgt | | | | A | B | C | D | E |
| Management doesn’t knowingly compromise patient safety: | Unit Mgt | A | B | C | D | | E | Hosp Mgt | | | | A | B | C | D | E |
| Management is doing a good job: | Unit Mgt | A | B | C | D | | E | Hosp Mgt | | | | A | B | C | D | E |
| Problem staff are dealt with constructively by our: | Unit Mgt | A | B | C | D | | E | Hosp Mgt | | | | A | B | C | D | E |
| I get adequate, timely information about events that might affect my work from: | Unit Mgt | A | B | C | D | | E | Hosp Mgt | | | | A | B | C | D | E |
| You can’t stop older people from falling. | | | | | | | | | | | | A | B | C | D | E |
| The levels of staffing in this ward are sufficient to handle the number of patients. | | | | | | | | | | | | A | B | C | D | E |
| It is my responsibility to update my patient’s falls risk status each shift if a fall and/or change in condition occurs. | | | | | | | | | | | | A | B | C | D | E |
| This hospital does a good job of training new staff. | | | | | | | | | | | | A | B | C | D | E |
| There are more important things I should do than falls prevention strategies for my high risk patients. | | | | | | | | | | | | A | B | C | D | E |
| All the necessary information for diagnostic and therapeutic decisions is routinely available to me. | | | | | | | | | | | | A | B | C | D | E |
| My supervisors have assisted when I raise problems about falls on my ward. | | | | | | | | | | | | A | B | C | D | E |
| Trainees in my discipline are adequately supervised. | | | | | | | | | | | | A | B | C | D | E |
| Positioning high falls risk patients in high visibility areas is an effective way to prevent them from falling. | | | | | | | | | | | | A | B | C | D | E |
| Targeted management programs for patients with delirium and confusion are used on my ward. | | | | | | | | | | | | A | B | C | D | E |
| High falls risk patients often have medication reviews to reduce their use of psychoactive drugs on my ward. | | | | | | | | | | | | A | B | C | D | E |
| There is strong leadership for falls prevention on my ward. | | | | | | | | | | | | A | B | C | D | E |

| **Please answer the following items with respect to your ward using the scale below.** | | | | | Strongly agree▼ | | | | | |
| --- | --- | --- | --- | --- | --- | --- | --- | --- | --- | --- |
|  | | | | Agree▼ | | | | | |  |
|  | | | Neutral▼ | | | | | |  |  |
|  | | Disagree▼ | | | | | |  |  |  |
|  | Strongly disagree▼ | | | | | |  |  |  |  |
| My supervisors are supportive of falls prevention activities on my ward. | | | | | | A | B | C | D | E |
| I experience good collaboration with other nurses on this ward. | | | | | | A | B | C | D | E |
| I am given useful feedback about whether I am using falls prevention strategies properly. | | | | | | A | B | C | D | E |
| This feedback helps me use falls prevention strategies more effectively. | | | | | | A | B | C | D | E |
| I experience good collaboration with staff physicians on this ward. | | | | | | A | B | C | D | E |
| I experience good collaboration with pharmacists on this ward | | | | | | A | B | C | D | E |
| If a patient has had a fall on the ward, this is always discussed at handover. | | | | | | A | B | C | D | E |
| I receive regular reminders to use falls prevention strategies. | | | | | | A | B | C | D | E |
| I receive useful support and training from falls prevention leaders. | | | | | | A | B | C | D | E |
| Falls risk assessment and prevention strategies have been incorporated into the ward’s standard processes. | | | | | | A | B | C | D | E |
| Falls prevention best practice guidelines are a useful resource. | | | | | | A | B | C | D | E |
| Use of ‘specials’ are an effective way of preventing patients from falling. | | | | | | A | B | C | D | E |
| In this ward it is difficult to discuss errors. | | | | | | A | B | C | D | E |
| I report all patient falls to the person in charge of my shift. | | | | | | A | B | C | D | E |
| I am encouraged by my colleagues to report any patient safety concerns I may have. | | | | | | A | B | C | D | E |
| I report all patient falls on the incident reporting system. | | | | | | A | B | C | D | E |
| The culture in this ward makes it easy to learn from errors of others. | | | | | | A | B | C | D | E |
| I document all patient falls in the patient file’s (medical records). | | | | | | A | B | C | D | E |
| Communication breakdowns that lead to delays in delivery of care are common | | | | | | A | B | C | D | E |
| Exercise programs are commonly used to reduce the risk of falls in high falls risk patients on my ward. | | | | | | A | B | C | D | E |
| Exercise programs are an effective way of preventing falls in high falls risk patients. | | | | | | A | B | C | D | E |
| Post falls management procedures are in place on my ward to ensure prompt identification of fall injuries. | | | | | | A | B | C | D | E |
| An active falls prevention leader is essential for falls prevention programs to be successful on my ward. | | | | | | A | B | C | D | E |

| A | B | C | D | E |
| --- | --- | --- | --- | --- |
| Strongly disagree | Disagree | Neutral | Agree | Strongly agree |

## Comments:

How do you think training in falls prevention could be improved at this hospital?

What are the best features of your current falls prevention program?

What features of your current falls prevention program need improvement?
