# Supplementary material for: Measuring safety climate in acute hospitals: Rasch analysis of the safety attitudes questionnaire
Source: BMC Health Serv Res. 2016 Sep 20;16:497. doi: 10.1186/s12913-016-1744-4 (PMC5029072; doi:10.1186/s12913-016-1744-4)
Supplement: Additional file 2: — Characteristics of nurses that completed the Safety Attitudes Questionnaire (SAQ) by hospital (DOCX 17 kb) [file 12913_2016_1744_MOESM2_ESM.docx]

Characteristics of nurses that completed the Safety Attitudes Questionnaire (SAQ) by hospital (*n*=420)

|  | **Hospital 1** | **Hospital 2** | **Hospital 3** | **Hospital 4** | **Hospital 5** | **Hospital 6** | **Total** |
| --- | --- | --- | --- | --- | --- | --- | --- |
|  | ***n* = 77** | ***n* = 52** | ***n* = 112** | ***n* = 67** | ***n* = 42** | ***n* = 70** | ***n* = 420** |
| Ward, *n* (%)  Medical  Surgical | 42 (54.5)  35 (45.5) | 34 (65.4)  18 (34.6) | 87 (77.7)  25 (22.3) | 41 (61.2)  26 (38.8) | 42 (100.0)  0 (0.0) | 70 (100.0)  0 (0.0) | 316 (75.2)  104 (24.8) |
| Experience, *n* (%)  <4 months  4-12 months  1-5 years  >5 years  Not recorded | 3 (3.9)  9 (11.7)  36 (46.8)  26 (33.8)  3 (3.9) | 7 (13.5)  11 (21.2)  9 (17.3)  22 (42.3)  3 (5.8) | 6 (5.4)  20 (17.4)  36 (32.1)  49 (43.8)  1 (0.9) | 2 (3.0)  11 (16.4)  28 (41.8)  24 (35.8)  2 (3.0) | 2 (4.8)  7 (16.7)  10 (23.8)  23 (54.8)  0 (0.0) | 3 (4.3)  15 (21.4)  23 (32.9)  25 (35.7)  4 (5.7) | 23 (5.5)  73 (17.4)  142 (33.8)  169 (40.2)  13 (3.1) |
| Qualification, *n* (%)  RN Div 1  EN Div 2  Other (e.g. PCA)  Not recorded | 68 (88.3)  3 (3.9)  1 (1.3)  5 (6.5) | 24 (46.2)  2 (3.8)  18 (34.6)  8 (15.4) | 91 (81.3)  18 (16.1)  0 (0.0)  3 (2.7) | 39 (58.2)  4 (6.0)  18 (26.9)  6 (9.0) | 31 (73.8)  10 (23.8)  0 (0.0)  1 (2.4) | 59 (84.3)  8 (11.4)  0 (0.0)  3 (4.3) | 312 (74.3)  45 (10.7)  37 (8.8)  26 (6.2) |
| Length of service, *n* (%)  <4 months  4-12 months  1-5 years  >5 years  Not recorded | 8 (10.4)  14 (18.2)  28 (36.4)  22 (28.6)  5 (6.5) | 7 (13.5)  10 (19.2)  6 (11.5)  22 (42.3)  7 (13.5) | 19 (17.0)  18 (16.1)  37 (33.0)  37 (33.0)  1 (0.9) | 9 (13.4)  13 (19.4)  28 (41.8)  15 (22.4)  2 (3.0) | 10 (23.8)  0 (0.0)  16 (38.1)  15 (35.7)  1 (2.4) | 14 (20.0)  7 (10.0)  22 (31.4)  24 (34.3)  3 (4.3) | 67 (16.0)  62 (14.8)  137 (32.6)  135 (32.1)  19 (4.5) |
| Number of shifts, *n* (%)  <1 shift/week  1 shift/week  2-4 shifts/week  5 shifts/week  Not recorded | 0 (0.0)  3 (3.9)  58 (75.3)  9 (11.7)  7 (19.1) | 0 (0.0)  1 (1.9)  14 (26.9)  35 (67.3)  2 (3.8) | 1 (0.9)  2 (1.8)  51 (45.5)  53 (47.3)  5 (4.5) | 0 (0.0)  0 (0.0)  13 (19.4)  52 (77.6)  2 (3.0) | 0 (0.0)  0 (0.0)  10 (23.8)  32 (76.2)  0 (0.0) | 2 (2.9)  3 (4.3)  29 (41.4)  35 (50.0)  1 (1.4) | 3 (0.7)  9 (2.1)  175 (41.7)  216 (51.4)  17 (4.0) |
| SAQ domain^§^, *n* (%)  Teamwork climate  Safety climate  Job satisfaction  Stress recognition  Perceptions of ward  management  Perceptions of hospital  management  Working conditions | 39 (51)  30 (39)  45 (58)  25 (32)  29 (38)  8 (10)  18 (23) | 16 (31)  6 (12)  19 (37)  26 (50)  4 (8)  3 (6)  7 (13) | 71 (63)  63 (56)  66 (59)  48 (43)  46 (41)  11 (10)  38 (34) | 37 (55)  30 (45)  35 (52)  30 (45)  24 (36)  7 (10)  23 (34) | 8 (19)  7 (17)  15 (36)  15 (36)  10 (24)  1 (2)  7 (17) | 41 (59)  35 (40)  44 (63)  23 (33)  27 (39)  9 (13)  21 (30) | 212 (51)  171 (41)  224 (53)  167 (40)  140 (33)  39 (9)  114 (27) |

RN, Registered Nurse; EN, Enrolled Nurse; PCA, Patient Care Assistants

^§^Number and percentage of nurses holding a positive attitude to each SAQ domain (> 75 out of 100)
