# Supplementary material for: Measuring safety climate in acute hospitals: Rasch analysis of the safety attitudes questionnaire
Source: BMC Health Serv Res. 2016 Sep 20;16:497. doi: 10.1186/s12913-016-1744-4 (PMC5029072; doi:10.1186/s12913-016-1744-4)
Supplement: Additional file 3: — Rasch item and fit statistics for the Safety Attitudes Questionnaire (SAQ) (DOCX 18 kb) [file 12913_2016_1744_MOESM3_ESM.docx]

Rasch item and fit statistics for the Safety Attitudes Questionnaire (SAQ)

| Domain | SAQ  Item | Item scores | Item statistics^ϯ^ | Fit statistics | | |
| --- | --- | --- | --- | --- | --- | --- |
|  |  | Mean (SD) | Location (SE) | Residuals* | χ^2§^ | F statistic^∞^ |
| Teamwork climate | 1 | 3.99 (0.92) | -0.15 (0.06) | -0.28 | 5.17 | 1.12 |
|  | 2 | 3.74 (1.09) | 0.11 (0.06) | **3.40** | **15.55** | 3.99 |
|  | 3 | 3.69 (0.92) | 0.25 (0.06) | 0.8 | 6.05 | 1.34 |
|  | 4 | 3.98 (0.83) | -0.31 (0.07) | -1.32 | **10.66** | 3.05 |
|  | 5 | 3.95 (0.88) | -0.14 (0.07) | -0.58 | 3.24 | 0.45 |
|  | 6 | 3.69 (0.86) | 0.24 (0.07) | 0.71 | 7.90 | 1.89 |
| Safety climate | 7 | 3.72 (0.86) | 0.11 (0.07) | 0.29 | 3.79 | 0.62 |
|  | 8 | 3.75 (0.82) | -0.10 (0.07) | 0.03 | 3.57 | 0.60 |
|  | 9 | 3.89 (0.78) | -0.29 (0.07) | -0.41 | 2.67 | 0.47 |
|  | 10 | 3.52 (0.91) | 0.34 (0.07) | -1.19 | 10.85 | 2.07 |
|  | 11 | 3.70 (1.00) | 0.18 (0.06) | 1.55 | 5.81 | 0.87 |
|  | 12 | 4.13 (0.80) | -0.43 (0.07) | -0.55 | 12.40 | 2.18 |
|  | 13 | 3.65 (0.91) | 0.19 (0.06) | 0.06 | 1.27 | 0.11 |
| Job satisfaction | 15 | 4.12 (0.76) | -0.69 (0.09) | 1.43 | 2.39 | 0.41 |
|  | 16 | 3.77 (0.99) | 0.47 (0.08) | -0.94 | 6.26 | 1.28 |
|  | 17 | 3.94 (0.86) | -0.08 (0.08) | -1.23 | **15.23** | 3.66 |
|  | 18 | 4.05 (0.81) | -0.79 (0.09) | **-3.29** | **17.41** | 5.27 |
|  | 19 | 3.48 (0.94) | 1.10 (0.08) | 1.81 | **17.87** | 3.06 |
| Stress recognition | 20 | 3.82 (1.08) | -0.35 (0.06) | 2.18 | 10.43 | 1.34 |
|  | 21 | 3.91 (1.03) | -0.54 (0.07) | -0.63 | **14.81** | 3.68 |
|  | 22 | 3.43 (1.18) | 0.19 (0.06) | -1.14 | 12.63 | 3.11 |
|  | 23 | 3.04 (1.21) | 0.70 (0.06) | 0.85 | 3.50 | 0.70 |
| Perceptions of ward management | 24 | 3.97 (0.91) | -0.50 (0.08) | **-3.41** | **21.39** | 5.70 |
|  | 25 | 3.60 (1.18) | 0.03 (0.06) | **3.54** | **33.51** | 5.88 |
|  | 26 | 3.95 (0.94) | -0.50 (0.07) | **-3.84** | **34.85** | 11.83 |
|  | 27 | 3.68 (0.94) | -0.03 (0.07) | 0.88 | 7.09 | 0.96 |
|  | 28 | 3.66 (0.94) | -0.12 (0.08) | -1.31 | **18.05** | 3.73 |
|  | 29 | 2.81 (1.12) | 1.11 (0.06) | **5.99** | **49.72** | 6.78 |
| Perceptions of hospital management | 24 | 3.03 (0.94) | 0.12 (0.07) | **-2.55** | **18.46** | 5.15 |
|  | 25 | 3.08 (0.95) | -0.24 (0.07) | 1.91 | **15.46** | 2.55 |
|  | 26 | 3.11 (0.95) | -0.09 (0.07) | **-3.73** | **30.40** | 10.18 |
|  | 27 | 3.15 (0.87) | -0.18 (0.07) | -1.07 | 5.95 | 1.39 |
|  | 28 | 3.06 (0.90) | 0.09 (0.07) | -1.03 | 8.26 | 1.84 |
|  | 29 | 2.81 (1.12) | 0.30 (0.05) | **7.39** | **74.67** | 10.83 |
| Working conditions | 30 | 3.31 (0.91) | 0.27 (0.07) | -1.00 | 3.83 | 0.85 |
|  | 31 | 3.46 (0.82) | -0.03 (0.08) | 1.19 | 5.15 | 1.03 |
|  | 32 | 3.54 (0.82) | -0.24 (0.08) | -0.62 | 5.19 | 1.30 |

^ϯ^Expressed in linear log-odds units (logits), with mean item location set at 0 for each scale.

*Log residuals summarise the deviation of observed from expected responses. Deviation from the recommended rang of +2.5, indicating item misfit, are in bold typeface.[18]

^§^χ^2^ values summarise the deviation of observed from expected responses across the sample. Higher absolute χ^2^ values represent larger deviations.

^∞^One way ANOVAs of deviations from model expectation across the sample.
